# Development and validation of a clinical prediction model for in-hospital mortality of severe pneumonia based on machine learning

**DOI:** 10.3389/fphar.2025.1660893

**Published:** 2025-11-26

**Authors:** Kai Xie, Xiajin Huang, Zhen Li, Wenjing Yin, Xiaoxuan He, Xinyu Miao, Haifeng Wang

**Affiliations:** 1 Department of National Regional TCM (Lung Disease) Diagnosis and Treatment Center, The First Affiliated Hospital of Henan University of Chinese Medicine, Zhengzhou, China; 2 Academy of Chinese Medical Sciences, Henan University of Chinese Medicine, Zhengzhou, China; 3 Co-construction Collaborative Innovation Center for Chinese Medicine and Respiratory Diseases by Henan & Education Ministry of P.R. China, Henan University of Chinese Medicine, Zhengzhou, China

**Keywords:** severe pneumonia, machine learning, prediction model, mortality, traditional Chinese medicine

## Abstract

**Objective:**

We aimed to develop an interpretable model to predict the mortality risk for severe pneumonia patients.

**Methods:**

The study retrospectively employed data from severe pneumonia patients at two hospitals as the training set for the model development. Patients with severe pneumonia admitted from the same two hospitals were prospectively included as the test set for the model evaluation. A total of 115 candidate features were extracted based on clinical relevance and existing literature. The least absolute shrinkage and selection operator (LASSO) regression was applied to select features for the establishment of five models: logistic regression (LR), support vector machine (SVM), decision tree (DT), random forest (RF) and extreme gradient boosting (XGBoost). The performance of the models was assessed from discrimination, calibration and clinical practicability. The optimal model was screened out, and SHapley Additive exPlanation (SHAP) method was used to explain.

**Results:**

A total of 323 eligible patients with severe pneumonia were enrolled, including 226 patients in the training set and 97 in test set. In comparison to the other four models, the XGBoost model demonstrated the third highest area under the receiver operating characteristic (AUROC), along with optimal calibration and clinical practicability. The SHAP value of the XGBoost model indicated that the application of retention catheterization was identified as the most important influential predictor in the model, followed by oral Chinese herbal decoction, blood urea nitrogen (BUN) level, age, application of tracheotomy, complication of septic shock, and TCM syndrome (pathogenic qi falling into and prostration syndrome).

**Conclusion:**

Older age, increased BUN level, complication of septic shock, tracheotomy application, retention catheterization application, oral Chinese herbal decoction, and TCM syndrome (pathogenic qi falling into and prostration syndrome) may be potential risk factors that affect mortality in severe pneumonia, while application of tracheotomy and oral Chinese herbal decoction were associated with reduced mortality. The XGBoost model exhibits superior overall performance in predicting hospital mortality risk for severe pneumonia, greater than traditional scoring systems such as Pneumonia Severity Index (PSI), Sequential Organ Failure Assessment (SOFA), and Acute Physiology and Chronic Health Evaluation II (APACHE II), which assists clinicians in prognostic assessment, resulting in improved therapeutic strategies and optimal resource allocation for patients.

## 1 Introduction

The 2019 Global Burden of Disease Study indicated that lower respiratory infections ranked as the fourth major cause of global mortality, resulting in over 2.49 million deaths, beaten only by newborn illnesses, ischemic heart disease, and stroke (GBD 2019 Diseases and Injuries Collaborators, 2020). Severe pneumonia is a frequently occurring life-threatening disease characterized by lower respiratory infection, with a high mortality, numerous complications, a poor prognosis, and a significant economic burden (Welte et al., 2012). Furthermore, it is a primary cause of ICU hospitalization and infection-related death around the world (Aliberti et al., 2016). In the United States, pneumonia causes 78% of infection-related deaths (Martin-Loeches et al., 2022). Despite continuous breakthroughs in therapy over the last few decades, severe pneumonia has always been linked with a significant death rate, ranging from 20% to more than 50% (Chen et al., 2020). Thus, the identification of early hospital mortality risk in patients with severe pneumonia is essential and may facilitate appropriate care and clinical decision support.

In recent years, identifying mortality risk factors in patients with severe pneumonia has emerged as the main study focus. Researchers have discovered several factors associated with mortality in patients with severe pneumonia, including high mean platelet volume levels (Chen et al., 2020), increased admission lactate (Huang et al., 2023), C-reactive protein (CRP)-to-albumin ratio (Zhang C. et al., 2023), admission interleukin (IL)-32 concentration (Espana et al., 2006), the Modified Nutrition Risk in Critically ill (mNUTRIC) score (Acehan et al., 2021), elevated stress hyperglycemia ratio (Miao et al., 2024), serum Krebs von den Lungen-6 (Lu et al., 2023), the ratio of total body water to fat-free mass (Tseng et al., 2024), thrombocytopenia (Dos Santos Medeiros et al., 2024), severe thinness (Body Mass Index <16 kg/m^2^) (Lee et al., 2015), and the presence of septic shock (Que et al., 2015). Nevertheless, these factors are comparatively singular and varied. Despite a systematic review that comprehensively analyze existing literature to identify mortality risk factors for severe pneumonia (Xie et al., 2024), there is an absence of precise prediction applicable to individual cases.

The clinical prediction model can estimate the probability of a specific individual currently suffering from a certain condition or experiencing a certain outcome in the future by assigning relative weights to each predictor variable and combining multiple predictor variables (Ranstam et al., 2016). There has been an increasing number of studies on prediction models worldwide. However, there is an absence of predictive models regarding the mortality risk associated with severe pneumonia that contain traditional Chinese medicine (TCM) characteristics, as well as inadequate comparisons among existing models; moreover, selection and consideration of predictive variables are insufficient. Hence, it is crucial to develop a comprehensive and systematic mortality risk prediction model for severe pneumonia containing TCM characteristics. Based on clinical needs, constructing prediction models can greatly promote the implementation of precision medicine, support thorough clinical diagnosis and evidence-based decision-making, and optimize public health resources allocation.

The advancement of electronic medical record systems has helped in the availability of substantial clinical data. Nonetheless, conventional logistic regression is incapable of managing complex clinical data (Li J. et al., 2021). Currently, artificial intelligence (AI) technology has achieved substantial breakthroughs, introducing novel techniques for data processing and extraction. Machine learning, a core component of AI, can autonomously develop data models, recognize complex data patterns, and predict results based on insights derived from computer algorithms (Bi et al., 2019). Due to the inherent capabilities of machine learning algorithms, an increasing number of researchers support the implementation of novel predictive models based on machine learning to facilitate suitable diagnosis and treatment, compared to conventional illness severity classification systems like the Sequential Organ Failure Assessment (SOFA) score or the Acute Physiology and Chronic Health Evaluation (APACHE) II score (Pirracchio et al., 2015).

Normal supervised machine learning classifiers possess distinct characteristics, and their performance is frequently dependent upon the attributes of the datasets being classified. Logistic regression (LR), support vector machine (SVM), decision tree (DT), random forest (RF), and extreme gradient boosting (XGBoost) are popular machine learning techniques; yet, their specific performance on severe pneumonia datasets remains ambiguous. Therefore, this study aimed to accurately, quickly, and comprehensively predict the individual mortality risk of patients with severe pneumonia and improve prognosis by establishing a mortality risk prediction model for severe pneumonia containing the characteristics of TCM using multiple machine learning algorithms.

## 2 Methods

### 2.1 Study design and population

This study was conducted to develop a model to predict hospital mortality in patients with severe pneumonia. A retrospective observational study in training set was designed to consecutively enroll patients in wards at the First Affiliated Hospital of Henan University of Chinese Medicine and Henan Provincial Hospital of Chinese Medicine from January 2008 to November 2021. The test set was consistent with patient source for the training set, but prospectively observational study from December 2021 to January 2024. The follow-up of all participants continued until discharge or death. This study was approved by the Ethics Committee of the First Affiliated Hospital of Henan University of Chinese Medicine (No. 2023HL-241-01). All patients or their legal guardians in the test set were asked to sign an informed consent form. However, due to the retrospective nature for the training set, the need to obtain the informed consent was waived by the Ethics Committee of the First Affiliated Hospital of Henan University of Chinese Medicine. This study complied with the principles defined in the Declaration of Helsinki and the International Conference on Harmonization-Good Clinical Practice guidelines.

### 2.2 Inclusion and exclusion criteria

The inclusion criteria for the training set were: (1) Participants must have a diagnosis of severe pneumonia in accordance with the guidelines established by the Respiratory Society of the Chinese Medical Association (Cao, 2016) or the Infectious Disease Society of America/American Thoracic Society (Metlay et al., 2019); (2) The diagnosis of TCM syndrome must adhere to the Traditional Chinese Medicine Diagnosis and Treatment Guidelines for Community-Acquired Pneumonia (2018 Revised Edition) published by the Chinese Medical Association (Yu et al., 2019); (3) There were no restrictions regarding the gender or comorbidities of the patients, except they had to be 18 years of age or older. The exclusion criteria were: (1) Numerous missing clinical data; (2) A hospital stay of fewer than 3 days.

The inclusion criteria for the test set were: (1) Participants must be diagnosed with severe pneumonia in accordance with the guidelines of the Respiratory Society of the Chinese Medical Association (Cao, 2016) or the Infectious Disease Society of America/American Thoracic Society (Metlay et al., 2019) and recruited within 3 days; (2) There were no restrictions regarding gender or comorbidities, provided participants were 18 years or older; (3) All patients, or their legal representatives in cases where they were unable to provide consent, were required to sign an informed consent form. Besides, individuals with dementia and other mental disorders were excluded.

We excluded patients with clearly diagnosed fungal and viral pneumonia, including severe Influenza A (H1N1), severe acute respiratory syndrome (SARS), or coronavirus disease 2019 (COVID-19) from both the training and test sets to improve homogeneity.

### 2.3 Outcome definition

The prediction outcome of this study was the probability of in-hospital mortality, defined as deaths during the current hospitalization period, including within 1 day after discharge.

### 2.4 Features extraction

A total of 115 candidate features were extracted based on clinical relevance and existing literature, encompassing the following categories: demographic characteristics (e.g., age, gender, solar term), clinical manifestations, admission risk factors (e.g., smoking history, recent hospitalization), comorbidities, complications, laboratory results, treatments during hospitalization (e.g., conventional medicine and operations), and TCM-specific variables (e.g., TCM syndromes, use of oral Chinese herbal decoction). This study incorporated the solar term as a predictive variable. This concept originates from the traditional Chinese lunisolar calendar, which partitions the year into 24 distinct periods, each reflecting a specific phase of climatic and environmental change. TCM theory posits that these rhythmic, seasonal transitions can modulate human physiological balance and susceptibility to disease. Therefore, including solar term allows the model to capture potential seasonality effects on the prognosis of severe pneumonia, which aligns with a holistic TCM approach to medicine. For laboratory results, the first value obtained within 24 h of admission was used. It should be noted that the variable oral Chinese herbal decoction was recorded as a binary feature indicating whether the patient was prescribed and administered any Chinese herbal decoction orally for at least five consecutive days during hospitalization. The oral Chinese herbal decoctions were not a fixed formula but were individualized prescriptions formulated by licensed TCM physicians based on real-time pattern differentiation according to the patient’s evolving clinical manifestations and TCM syndrome diagnosis. This reflects the standard, personalized approach of TCM clinical practice. As such, the specific herbal components and dosages varied across patient.

### 2.5 Missing data handling

Investigated and confirmed outliers and missing numbers in the original electronic medical records database. If verification or supplementation was not possible, consider the outlier as a missing value for processing. Variables with missing data over 25% were removed, while multiple imputation would be employed for those within 25%. Multiple imputation was performed using the mice package in R 4.3.2 software, with 5 imputations and predictive mean matching method. The outcome variable (in-hospital mortality) was included in the imputation model to ensure unbiased estimates.

### 2.6 Statistical analysis

Every statistical analysis and calculations were employed SPSS 26.0 or R 4.3.2 software. The categorical variables expressed as total numbers and percentages, and the χ2 test or Fisher exact test (expected frequency <10) was employed to compare group differences. The normality test was performed on all continuous variables to ascertain if the data adhered to a normal distribution, mostly using the Shapiro Wilk or Kolmogorov Smirnov tests in conjunction with histograms. If the data adhered to a normal distribution, represented as mean ( $\bar{x}$ ) ± standard deviation (SD), an t-test was employed to assess group differences; conversely, it was denoted by the median and interquartile range (IQR), and applied the Wilcoxon rank sum test.

### 2.7 Features selection

Patients with severe pneumonia were categorized into non-survivor and survivor groups based on in-hospital mortality, and characteristics were presented and compared between the groups. The 115 features collected from the training set underwent statistical analysis to identify variables with significant differences between the non-survivor and survivor groups. Additionally, to prevent overfitting, the Least Absolute Shrinkage and Selection Operator (LASSO) using the glmnet package in R 4.3.2 software was employed with 10-fold cross-validation to identify and refine candidate predictors (Muthukrishnan and Rohini, 2016). The simplest subset of predictive factors was chosen to identify the independent features for inclusion in the in-hospital mortality risk prediction model for severe pneumonia.

### 2.8 Model development and performance evaluation

Five machine learning algorithms were employed to develop predictive models: LR, SVM, DT, RF, and XGBoost in R version 4.3.2. To ensure a fair and transparent baseline comparison of the algorithms’ out-of-the-box performance, all models were run using their respective package’s default hyperparameters. A detailed parameter table has been added as Supplementary Table S1. For the LR model, which assumes independence among predictors, we assessed multicollinearity using the variance inflation factor (VIF) implemented in the car package R version 4.3.2. A VIF value exceeding 10 was considered indicative of severe multicollinearity, following common statistical conventions (O’brien, 2007). The discrimination of each model was assessed using the area under the receiver operating characteristic curve (AUROC) and confusion matrix. We incorporated balanced accuracy and matthew correlation coefficient (MCC), as recommended for imbalanced datasets. Besides, DeLong test was used to compare AUC values and further evaluate the differences in predictive performance between models. The calibration curve assessed the calibration; furthermore, to test the clinical applicability for decision-making by estimating the net benefit at various threshold probabilities, decision curve analysis (DCA) was conducted (Van Calster et al., 2018). The performance of our optimal machine learning model was benchmarked against traditional severity-of-illness scores, namely the Pneumonia Severity Index (PSI), Sequential Organ Failure Assessment (SOFA), and Acute Physiology and Chronic Health Evaluation II (APACHE II). The traditional severity scores of PSI, SOFA, and APACHE II were calculated according to their standard, widely-used definitions: PSI as defined by Fine et al. (Fine et al., 1997), SOFA as per the original consensus conference definition (Vincent et al., 1996), and APACHE II following Knaus et al. (Knaus et al., 1985). It was important to note that these scores were not used as input features for training any of the machine learning models. They were calculated retrospectively after model development and served as independent, external comparators to evaluate the relative predictive gain of our new model.

## 3 Results

### 3.1 Overall flow

**FIGURE 1:**
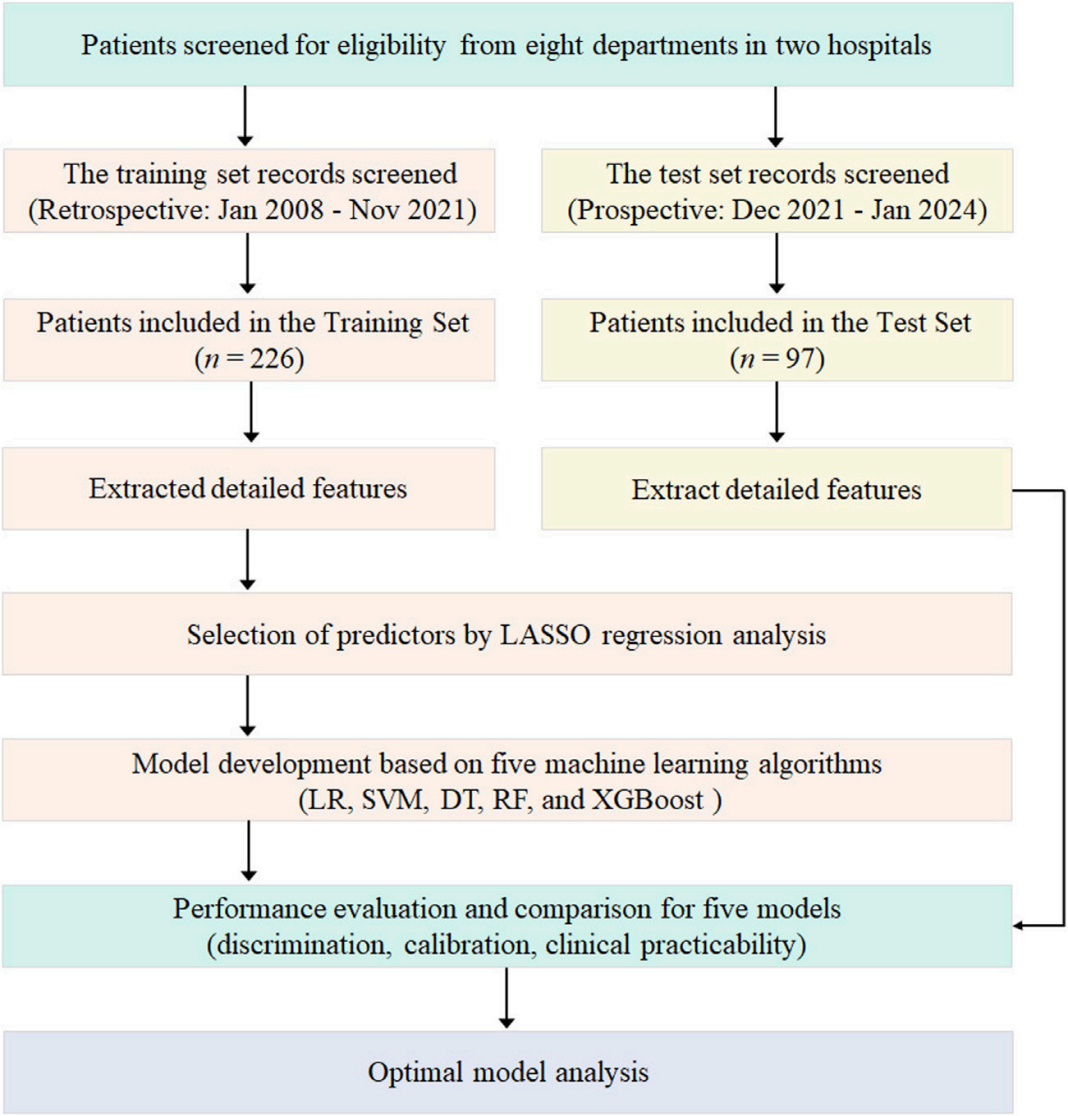
Flow chart of the study procedure. This chart illustrates the key stages of the study, including patient cohort formation, feature extraction, predictor selection, model development, and evaluation. LASSO, Least Absolute Shrinkage and Selection Operator; LR, Logistic Regression; SVM, Support Vector Machine; DT, Decision Tree; RF, Random Forest; XGBoost, Extreme Gradient Boosting.

The schematic flow of this study was shown in Figure 1. Patients were screened from eight departments across two affiliated hospitals. A retrospective training set (January 2008 to November 2021) and a prospective test set (December 2021 to January 2024) were established. Detailed features were extracted for all included patients. Predictors were then selected from these features using LASSO regression analysis. Subsequently, five machine learning models (LR, SVM, DT, RF, and XGBoost) were developed and their performance was comprehensively evaluated and compared in terms of discrimination, calibration, and clinical practicability to identify the optimal model.

### 3.2 Basic features

In the training set, 170 patients (75.2%) were from the First Affiliated Hospital of Henan University of Chinese Medicine, and 56 (24.8%) patients were from Henan Provincial Hospital of Chinese Medicine. The prospective test set comprised 57 patients (58.8%) from the First Affiliated Hospital of Henan University of Chinese Medicine and 40 (41.2%) from Henan Provincial Hospital of Chinese Medicine. A total of 226 adult patients diagnosed with severe pneumonia were included into the final training set for this study, while 97 in the test set. The in-hospital mortality for severe pneumonia was 23.82% in the training set and 29.89% in the test set. No significant difference in mortality was seen between the training and test sets (*P* > 0.05). The comparison of features between the training set and the test set was presented in Table 1. The training set mainly occurred during the Lesser Cold, Greater Cold, Grain in Beard, and Winter Solstice solar periods, whereas the test set was primarily linked with the Winter Solstice and Lesser Cold. The distribution between the two datasets was statistically significant (*P* = 0.034). In the training set, the history of alcohol consumption, the presence of pleural effusion comorbidity, the use of three or more antibiotics, the frequency of quinolone antibiotics, as well as hematocrit, ALT, and D-dimer levels, and the duration of mask oxygen therapy were all significantly raised compared to the test set (*P* < 0.05). The comorbidity of gastrointestinal bleeding and the levels of PCT, myohemoglobin, and PaO_2_ were considerably reduced compared to the test set (*P* < 0.05). No substantial difference was observed in other features (*P* > 0.05).

**TABLE 1:** Features comparison between the training and test set.

Feature	Training set (*n* = 226)	Test set (*n* = 97)	*P value*
Age	72 (63–82)	76 (65–84)	0.155
Male	158 (69.91%)	60 (61.86%)	0.157
Nationalitiy			1.000
Han	224 (99.12%)	96 (98.97%)	
Others	2 (0.88%)	1 (1.03%)	
Solar term			0.034*
Lesser cold	16 (7.08%)	11 (11.34%)	
Greater cold	16 (7.08%)	3 (3.09%)	
The beginning of spring	9 (3.98%)	7 (7.22%)	
Rain water	10 (4.42%)	4 (4.12%)	
Insects awaken	9 (3.98%)	1 (1.03%)	
The spring equinox	6 (2.65%)	7 (7.22%)	
Pure brightness	7 (3.10%)	3 (3.09%)	
Grain rain	10 (4.42%)	5 (5.15%)	
The beginning of summer	7 (3.10%)	6 (6.19%)	
Lesser fullness of grain	5 (2.21%)	5 (5.15%)	
Grain in beard	16 (7.08%)	4 (4.12%)	
The summer solstice	6 (2.65%)	2 (2.06%)	
Lesser heat	11 (4.87%)	2 (2.06%)	
Greater heat	11 (4.87%)	2 (2.06%)	
The beginning of autumn	6 (2.65%)	4 (4.12%)	
The end of heat	7 (3.10%)	7 (7.22%)	
White dew	5 (2.21%)	1 (1.03%)	
The autumn equinox	11 (4.87%)	4 (4.12%)	
Cold dew	8 (3.54%)	0 (0.00%)	
Frost’s descent	8 (3.54%)	1 (1.03%)	
The beginning of winter	11 (4.87%)	0 (0.00%)	
Lesser snow	7 (3.10%)	1 (1.03%)	
Greater snow	10 (4.42%)	4 (4.12%)	
The winter solstice	14 (6.19%)	13 (13.40%)	
Vital signs			
Body temperature (°C)	36.9 (36.5–38.23)	36.8 (36.5–37.8)	0.226
Respiratory rate (breaths/min)	22 (20–27)	22 (20–26)	0.770
Heart rate (beats/min)	97.5 (80–113)	89 (80–107)	0.330
Systolic pressure (mmHg)	126 (114–140)	123 (113.5–135.5)	0.180
Diastolic pressure (mmHg)	76 (69–84)	75 (66–81)	0.224
Risk factors before admission			
Allergic history	27 (11.95%)	17 (17.53%)	0.180
Smoking history	46 (20.35%)	14 (14.43%)	0.210
Alcohol consumption history	35 (15.49%)	7 (7.22%)	0.043*
Fracture history	25 (11.06%)	11 (11.34%)	0.942
Surgery history	83 (36.73%)	30 (30.9%)	0.317
Long-term bed rest history	97 (42.92%)	46 (47.42%)	0.455
Hospitalization within 90 days	137 (60.62%)	60 (61.86%)	0.835
ICU admission within 90 days	41 (18.14%)	17 (17.53%)	0.895
Intravenous antibiotics within 30 days	112 (49.56%)	58 (59.79%)	0.091
Dialysis within 30 days	7 (3.10%)	3 (3.09%)	1.000
Comorbidities			
Hypertension	117 (51.77%)	50 (51.55%)	0.971
Diabetes	68 (30.09%)	33 (34.02%)	0.971
Chronic bronchitis	28 (12.39%)	4 (4.12%)	0.023
COPD	33 (14.60%)	12 (12.37%)	0.596
Pulmonary fibrosis	27 (11.95%)	16 (16.49%)	0.270
Bronchiectasis	7 (3.10%)	3 (3.09%)	1.000
Asthma	10 (4.42%)	1 (1.03%)	1.000
Old pulmonary tuberculosis	9 (3.98%)	2 (2.06%)	0.591
Pulmonary abscess	2 (0.88%)	0 (0.00%)	1.000
Pulmonary heart disease	10 (4.42%)	1 (1.03%)	0.227
Arrhythmia	54 (23.89%)	33 (34.02%)	0.081
Cardiac insufficiency	22 (9.73%)	14 (14.43%)	0.219
Chronic heart failure	23 (10.18%)	14 (14.43%)	0.271
Parkinson’s disease	10 (4.42%)	4 (4.12%)	1.000
Cerebral infarction	87 (38.50%)	35 (36.08%)	0.682
Hematencephalon	23 (10.18%)	8 (8.25%)	0.589
Chronic gastritis	12 (5.31%)	3 (3.09%)	0.562
Gastrointestinal bleeding	8 (3.54%)	9 (9.28%)	0.034*
Chronic viral hepatitis	12 (5.31%)	3 (3.09%)	0.562
Liver cirrhosis	7 (3.10%)	2 (2.06%)	0.881
Chronic renal insufficiency	9 (3.98%)	8 (8.25%)	0.116
Chronic renal failure	8 (3.54%)	7 (7.22%)	0.250
Cancer	14 (6.19%)	9 (9.28%)	0.323
Lumbar disease	15 (6.64%)	2 (2.06%)	0.091
Neck disease	6 (2.65%)	3 (3.09%)	1.000
Complications			
Acid base disturbance	124 (54.87%)	56 (57.73%)	0.635
Electrolyte imbalance	145 (64.16%)	68 (70.10%)	0.301
Anemia	135 (59.73%)	66 (68.04%)	0.158
Hypoproteinemia	189 (83.63%)	89 (91.75%)	0.053
Pleural effusion	196 (86.73%)	64 (65.98%)	<0.001*
Acute myocardial infarction	7 (3.10%)	3 (3.09%)	1.000
Acute heart failure	19 (8.41%)	7 (7.22%)	0.718
Acute kidney injury	19 (8.41%)	8 (8.25%)	0.962
Acute liver injury	20 (8.85%)	12 (12.37%)	0.962
Hypovolemic shock	4 (1.77%)	2 (2.06%)	1.000
Septic shock	29 (12.83%)	16 (16.49%)	0.384
Cardiac shock	6 (2.65%)	0 (0.00%)	0.242
Laboratory results			
WBC (×10^9^/L)	8.8 (6.68–12.58)	9.7 (6.89–14.09)	0.299
RBC (×10^12^/L)	3.82 ± 0.83	3.62 ± 0.88	0.058
Hemoglobin (g/L)	117 (99.75–132.25)	112 (91.5–126.5)	0.163
Hematokrit (%)	36 (30.7–40.4)	33.3 (28.15–38.25)	0.011*
Platelet count (×10^9^/L)	191 (133–246)	206 (131–275)	0.161
NEUT%	86.15 (78.23–90.6)	85.9 (77.7–90.5)	0.963
LY%	9.1 (5.08–14.64)	8.4 (5.05–15.2)	0.961
CRP (mg/L)	81.21 (33.03–161)	83.2 (30.88–157.9)	0.953
PCT (μg/L)	0.36 (0.12–1.3)	0.4 (0.2–1.92)	0.022*
Total bilirubin (μmol/L)	13.2 (9.38–19.6)	12.3 (7.7–18.5)	0.156
Total protein (g/L)	59.38 ± 9.71	57.84 ± 7.49	0.123
Albumin (g/L)	31.5 ± 5.45	30.38 ± 4.95	0.082
ALT (U/L)	22.1 (13.45–37.4)	17 (11.15–27.25)	0.017*
AST (U/L)	26.9 (17.38–44.55)	24 (17.7–46.45)	0.313
BUN (mmol/L)	7.39 (5.09–12.91)	9.22 (5.06–15.56)	0.209
Scr (μmol/L)	70.55 (52.05–106.33)	60 (46.05–109.55)	0.177
Potassium (mmol/L)	4.17 ± 0.75	4.07 ± 0.68	0.275
Sodium (mmol/L)	137.6 (134–141)	137 (134–141.4)	0.866
Troponin (ng/mL)	0.05 (0.02–0.1)	0.05 (0.03–0.09)	0.758
Myohemoglobin (ng/mL)	40.74 (28.38–69.83)	45.7 (44.7–73.6)	0.000*
PT (s)	13.2 (11.9–14.8)	13.60 (11.95–15.40)	0.312
APTT (s)	33.20 (28.70–39.70)	31.90 (28.61–39.25)	0.393
Fibrinogen (g/L)	5.16 (3.79–6.49)	5.19 (3.81–6.55)	0.735
D-dimer (μg/mL)	2.29 (1.18–4.18)	1.87 (1.04–2.77)	0.009*
BNP (pg/mL)	223 (81.57–814.5)	223 (119–635.5)	0.332
Arterial blood PH	7.44 (7.4–7.48)	7.45 (7.38–7.48)	0.364
PaO_2_ (mmHg)	61.4 (55–75.65)	62.7 (58.9–80)	0.033*
PaCO_2_ (mmHg)	32.95 (28.93–39.08)	33 (31.1–36.9)	0.374
PaO_2_/FiO_2_	229.5 (190.25–270)	230 (145.5–282)	0.931
Application of conventional medicine			
Glucocorticoids	127 (56.19%)	58 (59.79%)	0.549
Number of antibiotics ≥3	171 (75.66%)	58 (59.79%)	0.004*
Beta-lactam antibiotics	222 (98.23%)	92 (94.85%)	0.185
Quinolone antibiotics	163 (72.12%)	44 (45.36%)	<0.001*
Aminoglycoside antibiotics	26 (11.50%)	8 (8.25%)	0.382
Macrolide antibiotics	37 (16.37%)	15 (15.46%)	0.839
Tetracycline antibiotics	40 (17.70%)	22 (22.68%)	0.297
Sulfonamide antibiotics	1 (0.44%)	0 (0.00%)	1.000
Antifungal drug	72 (31.86%)	37 (38.14%)	0.273
Immunosuppressant	9 (3.98%)	2 (2.06%)	0.591
Conventional operation			
Fiber bronchoscope	107 (47.35%)	45 (46.39%)	0.875
Transfusion	52 (23.01%)	28 (28.87%)	0.264
Hemodialysis	12 (5.31%)	9 (9.28%)	0.185
ECMO	2 (0.88%)	1 (1.03%)	1.000
Tracheotomy	33 (14.60%)	17 (17.53%)	0.505
Retention catheterization	129 (57.08%)	60 (61.86%)	0.425
Gastric intubation	123 (54.42%)	57 (58.76%)	0.472
Deep vein catheterization	114 (50.44%)	49 (50.52%)	0.990
Days of nasal tube oxygen	2 (0–12)	0 (0–8)	0.077
Days of mask oxygen days	0 (0–0)	0 (0–0)	0.009*
Days of non-invasive mechanical ventilation	0 (0–3)	0 (0–2)	0.505
Days of invasive mechanical ventilation	0 (0–7)	0 (0–6)	0.796
Days of mechanical ventilation	3 (0–11)	3 (0–10)	0.871
TCM or TCM appropriate technology			
Oral Chinese herbal decoction	166 (73.45%)	61 (62.89%)	0.064
Chinese patent medicine injection	181 (80.09%)	84 (86.60%)	0.162
TCM appropriate technology	190 (84.07%)	78 (80.41%)	0.423
TCM syndrome			
Phlegm-heat obstructing lung syndrome	78 (34.51%)	41 (42.27%)	0.354
Phlegm turbidity obstructing lung syndrome	43 (19.03%)	22 (22.68%)	
Deficiency of both qi and yin syndrome	23 (10.18%)	6 (6.19%)	
Lung-spleen qi deficiency syndrome	16 (7.08%)	11 (11.34%)	
Lung-spleen qi deficiency combined with phlegm turbidity obstructing lung syndrome	13 (5.75%)	3 (3.09%)	
Phlegm turbidity obstructing lung combined with stagnation of blood syndrome	9 (3.98%)	0 (0.00%)	
Deficiency of both qi and yin combined with phlegm turbidity obstructing lung syndrome	8 (3.54%)	3 (3.09%)	
Pathogenic qi falling into and prostration syndrome	7 (3.10%)	3 (3.09%)	
Invasion of pericardium by heat syndrome	6 (2.65%)	2 (2.06%)	
Phlegm-heat obstructing lung combined with stagnation of blood syndrome	6 (2.65%)	0 (0.00%)	
Deficiency of both qi and yin combined with phlegm-heat obstructing lung syndrome	6 (2.65%)	3 (3.09%)	
Lung-spleen qi deficiency combined with phlegm-heat obstructing lung syndrome	5 (2.21%)	1 (1.03%)	
Lung-spleen qi deficiency combined with stagnation of blood syndrome	4 (1.77%)	0 (0.00%)	
Stagnation of blood syndrome	1 (0.44%)	1 (1.03%)	
Deficiency of both qi and yin combined with stagnation of blood syndrome	1 (0.44%)	1 (1.03%)	
Others			
Multi-drug resistant bacterial infection	69 (30.53%)	31 (32.96%)	0.896
Total hospitalization days	16 (11–27)	15 (9.5–24.5)	0.266
Days of ICU stay	0 (0–7)	0 (0–7)	0.710

Data are presented as mean ± standard deviation for normally distributed continuous variables, and median (interquartile range) for non-normally distributed variables.

### 3.3 Features selection

A comparison of all 115 features was provided in Supplementary Table S2. Table 2 summarized a total of 38 features that demonstrated a statistically significant difference (*P* < 0.05) between non-survivors and survivors in the training set, which might be the potential risk factors for death in patients with severe pneumonia.

**TABLE 2:** Features comparison with significant differences between the non-survivors and the survivors for severe pneumonia patients in the training set.

Feature	Non-survivors (*n* = 64)	Survivors (*n* = 162)	*P value*
Age	78.5 (69.25–85.75)	70.5 (58.75–79.25)	<0.001*
Risk factors before admission			
Fracture history	12 (18.75%)	13 (8.02%)	0.032*
Long-term bed rest history	35 (54.69%)	62 (38.27%)	0.026*
Intravenous antibiotics within 30 days	23 (35.94%)	89 (54.94%)	0.010*
Comorbidities			
Cardiac insufficiency	12 (18.75%)	10 (6.17%)	0.006*
Cerebral infarction	35 (54.69%)	52 (32.10%)	0.002*
Gastrointestinal bleeding	6 (9.38%)	2 (1.23%)	0.010*
Cancer	9 (14.06%)	5 (3.09%)	0.005*
Complications			
Electrolyte imbalance	48 (75.00%)	97 (59.88%)	0.045*
Anemia	46 (71.88%)	89 (54.94%)	0.024*
Hypoproteinemia	59 (92.19%)	130 (80.25%)	0.029*
Pleural effusion	62 (96.88%)	134 (82.72%)	0.005*
Septic shock	18 (28.13%)	11 (6.79%)	<0.001*
Laboratory results			
Hematokrit (%)	33.9 (28.75–38.38)	36.6 (31.35–40.73)	0.021*
NEUT%	88.7 (82.95–93.2)	84.2 (76.28–89.83)	0.004*
PCT (μg/L)	0.62 (0.36–2.95)	0.35 (0.1–0.77)	<0.001*
Total protein (g/L)	56.71 ± 11.19	60.44 ± 8.88	0.019*
Albumin (g/L)	29.75 ± 6.3	32.19 ± 4.93	0.007*
BUN (mmol/L)	11.85 (7.4–17.52)	6.48 (4.63–10.38)	<0.001*
Scr (μmol/L)	81.75 (56–134.58)	65.5 (50.15–94.7)	0.021*
Troponin (ng/mL)	0.05 (0.05–0.19)	0.05 (0.01–0.07)	<0.001*
Myohemoglobin (ng/mL)	40.74 (40.74–78)	40.74 (21.1–69.83)	0.022*
Fibrinogen (g/L)	4.62 (3.03–5.8)	5.37 (4.1–6.56)	0.013*
D-dimer (μg/mL)	2.93 (1.79–5.06)	2.11 (1.02–3.79)	0.007*
Arterial blood PH	7.43 (7.36–7.46)	7.44 (7.42–7.48)	0.008*
Application of conventional medicine			
Antifungal drug	27 (42.19%)	45 (27.78%)	0.041*
Conventional operation			
Transfusion	21 (32.81%)	31 (19.14%)	0.035*
Tracheotomy	3 (4.69%)	30 (18.52%)	0.008*
Retention catheterization	56 (87.50%)	73 (45.06%)	<0.001*
Gastric intubation	45 (70.31%)	78 (48.15%)	0.003*
Deep vein catheterization	39 (60.94%)	75 (46.30%)	0.047*
Days of nasal tube oxygen	0 (0–8.5)	4 (0–14)	0.019*
Days of invasive mechanical ventilation	1.5 (0–8)	0 (0–4)	<0.001*
Days of mechanical ventilation	6.5 (1–11)	0 (0–11)	0.005*
TCM or TCM appropriate technology			
Oral Chinese herbal decoction	27 (42.19%)	139 (85.80%)	<0.001*
TCM syndrome			<0.001*
Phlegm-heat obstructing lung syndrome	13 (20.31%)	65 (40.12%)	
Phlegm turbidity obstructing lung syndrome	17 (26.56%)	26 (16.05%)	
Deficiency of both qi and yin syndrome	9 (14.06%)	14 (8.64%)	
Lung-spleen qi deficiency syndrome	6 (9.38%)	10 (6.17%)	
Lung-spleen qi deficiency combined with phlegm turbidity obstructing lung syndrome	2 (3.13%)	11 (6.79%)	
Phlegm turbidity obstructing lung combined with stagnation of blood syndrome	2 (3.13%)	7 (4.32%)	
Deficiency of both qi and yin combined with phlegm turbidity obstructing lung syndrome	1 (1.56%)	7 (4.32%)	
Pathogenic qi falling into and prostration syndrome	6 (9.38%)	1 (0.62%)	
Invasion of pericardium by heat syndrome	0 (0.00%)	6 (3.70%)	
Phlegm-heat obstructing lung combined with stagnation of blood syndrome	1 (1.56%)	5 (3.09%)	
Deficiency of both qi and yin combined with phlegm-heat obstructing lung syndrome	2 (3.13%)	4 (2.47%)	
Lung-spleen qi deficiency combined with phlegm-heat obstructing lung syndrome	1 (1.56%)	4 (2.47%)	
Lung-spleen qi deficiency combined with stagnation of blood syndrome	2 (3.13%)	2 (1.23%)	
Stagnation of blood syndrome	1 (1.56%)	0 (0.00%)	
Deficiency of both qi and yin combined with stagnation of blood syndrome	1 (1.56%)	0 (0.00%)	
Others			
Total hospitalization days	12 (8.25–23)	17 (13–27)	0.001*
Days of ICU stay	4 (0–10.5)	0 (0–4.25)	<0.001*

**FIGURE 2:**
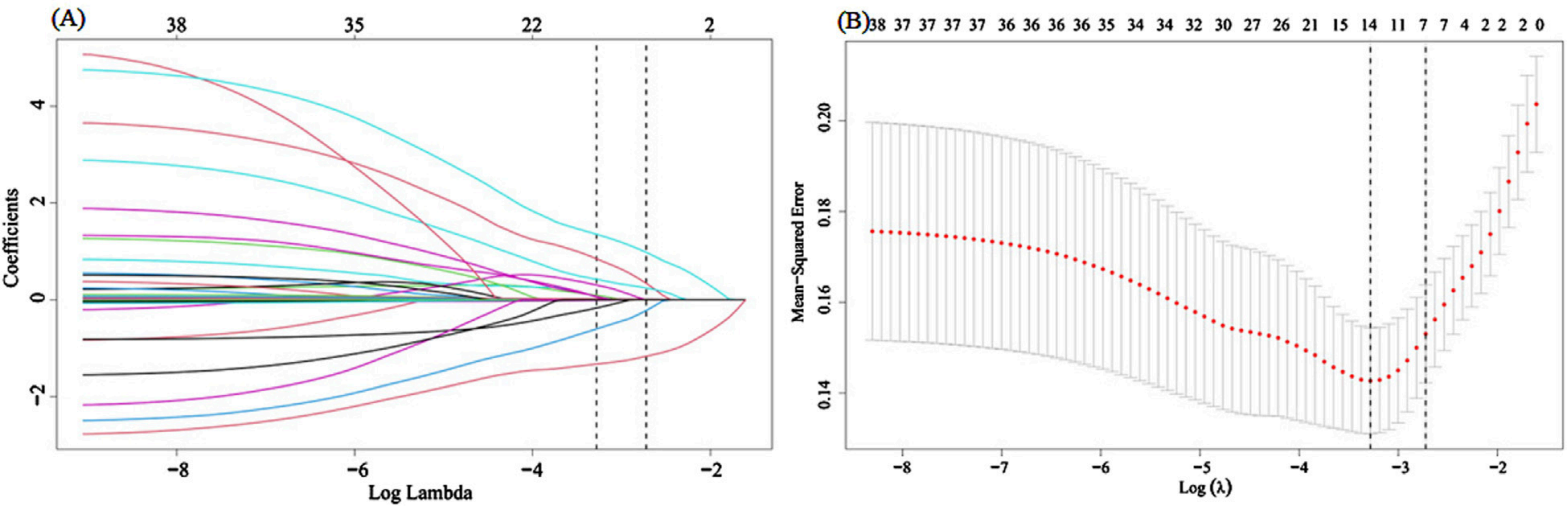
Selection of predictors by LASSO regression analysis with 10-fold cross-validation. **(A)** A coefficient profile plot was generated against the log (lambda) sequence. **(B)** The selection of the parameter (lambda) of deviance in LASSO regression was tuned according to the minimum and 1se criterion, indicated by the left and right dotted lines, respectively.

The LASSO regression identified 7 predictors from above 38 possible risk factors for 226 patients in the training set according to the lambda.1se criterion for predictor selection, which selects the most regularized model whose performance is within one standard error of the optimal lambda (Figure 2). All of the 7 predictors including age, TCM syndrome (pathogenic qi falling into and prostration syndrome), complication of septic shock, BUN level, tracheotomy application, retention catheterization application, and oral Chinese herbal decoction entered the final LR, SVM, DT, RF and XGBoost models.

### 3.4 Model development, evaluation and comparison

#### 3.4.1 Discrimination

**FIGURE 3:**
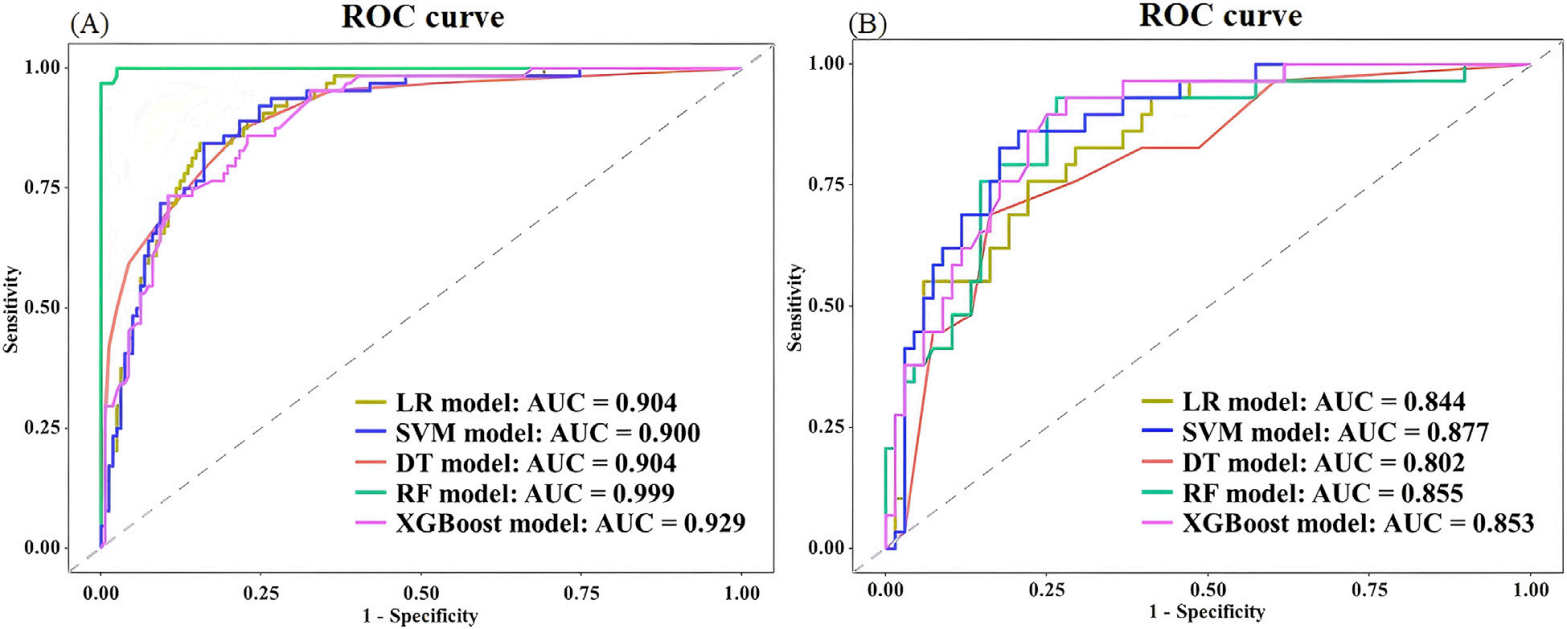
Comparison of ROC curves between different models in the training set **(A)** and test set **(B)**.

**FIGURE 4:**
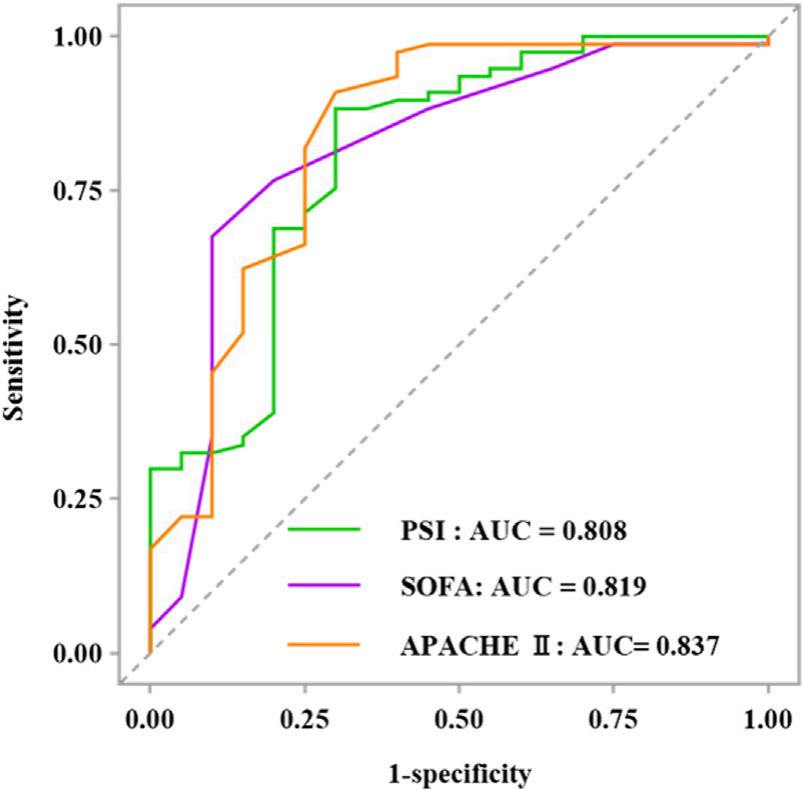
Comparison of ROC curves between different scoring systems of the PSI, SOFA, and APACHE Ⅱ in the test set.

**FIGURE 5:**
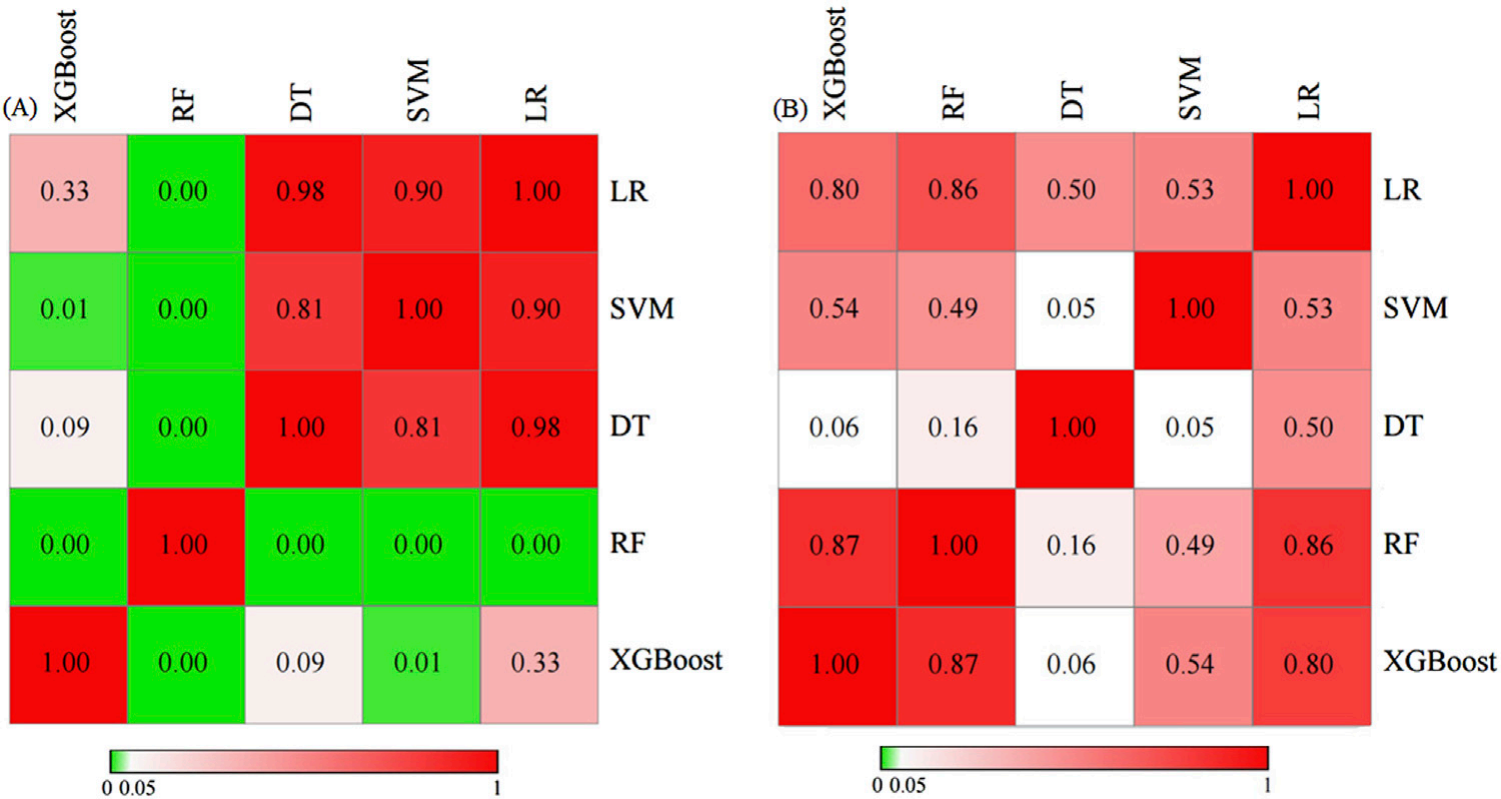
Comparison of AUC values by DeLong test between different models in the training set **(A)** and test set **(B)**.

The RF model demonstrated superior performance in the training set, attaining an accuracy of 0.982, a recall of 1.000, a precision of 0.941, an F1 score of 0.970, and an AUC of 0.999. The excessive high value of the indicators might be related to the overfitting of this model. The SVM indicators exhibited the lowest values among the five models, with an accuracy of 0.159, recall of 0.156, precision of 0.068, F1 score of 0.095, and AUC of 0.900. Moreover, the AUC of all five prediction models over 0.9, signifying a strong fitting performance in the training set. In the test set, the SVM model showed significantly inferior accuracy, recall, precision, and F1 score compared to other models, yet had the best AUC. The RF model revealed superior performance in accuracy, recall, precision, and F1 score metrics, with an AUC value ranking second only to the SVM model among the five models. The XGBoost model had the third best AUROC (0.853). The predicted value of the XGBoost model exceeded that of the PSI, SOFA, and APACHE II scoring systems, which showed AUC values of 0.808, 0.819, and 0.837, respectively. The RF model achieved the highest balanced accuracy and MCC both in the training and test sets, further confirming its robust and balanced performance in predicting both mortality and survival cases. The XGBoost model demonstrated a balanced accuracy of 0.757 and MCC of 0.472 on the test set. In contrast, the SVM model showed notably lower values in these metrics, aligning with its poor performance observed in other measures. The comprehensive results of the discrimination among the five models presented in Table 3, while the ROC curves are illustrated in Figures 3, 4. The DeLong test showed that there were significant differences in AUC between the RF model and others in the training set, also between the XGBoost and SVM models (*P* < 0.05); but there was no significant difference among the models in the test set (*P* > 0.05). The results of DeLong test illustrated in Figure 5.

**TABLE 3:** Comparing the discrimination of the five severe pneumonia hospital mortality prediction models.

Model	Training set (*n* = 226)							Test set (*n* = 97)						
	Accuracy	Recall	Precision	F1 score	AUC	Balanced accuracy	MCC	Accuracy	Recall	Precision	F1 score	AUC	Balanced accuracy	MCC
LR	0.845	0.844	0.684	0.755	0.904	0.845	0.674	0.732	0.793	0.535	0.639	0.844	0.749	0.460
SVM	0.159	0.156	0.068	0.095	0.900	0.158	−0.267	0.247	0.103	0.060	0.076	0.877	0.206	−0.538
DT	0.854	0.844	0.594	0.697	0.904	0.850	0.621	0.753	0.609	0.483	0.539	0.802	0.703	0.377
RF	0.982	1.000	0.941	0.970	0.999	0.988	0.958	0.773	0.793	0.590	0.677	0.855	0.779	0.521
XGBoost	0.788	0.906	0.580	0.707	0.929	0.824	0.587	0.701	0.897	0.500	0.642	0.853	0.757	0.472

#### 3.4.2 Calibration

**FIGURE 6:**
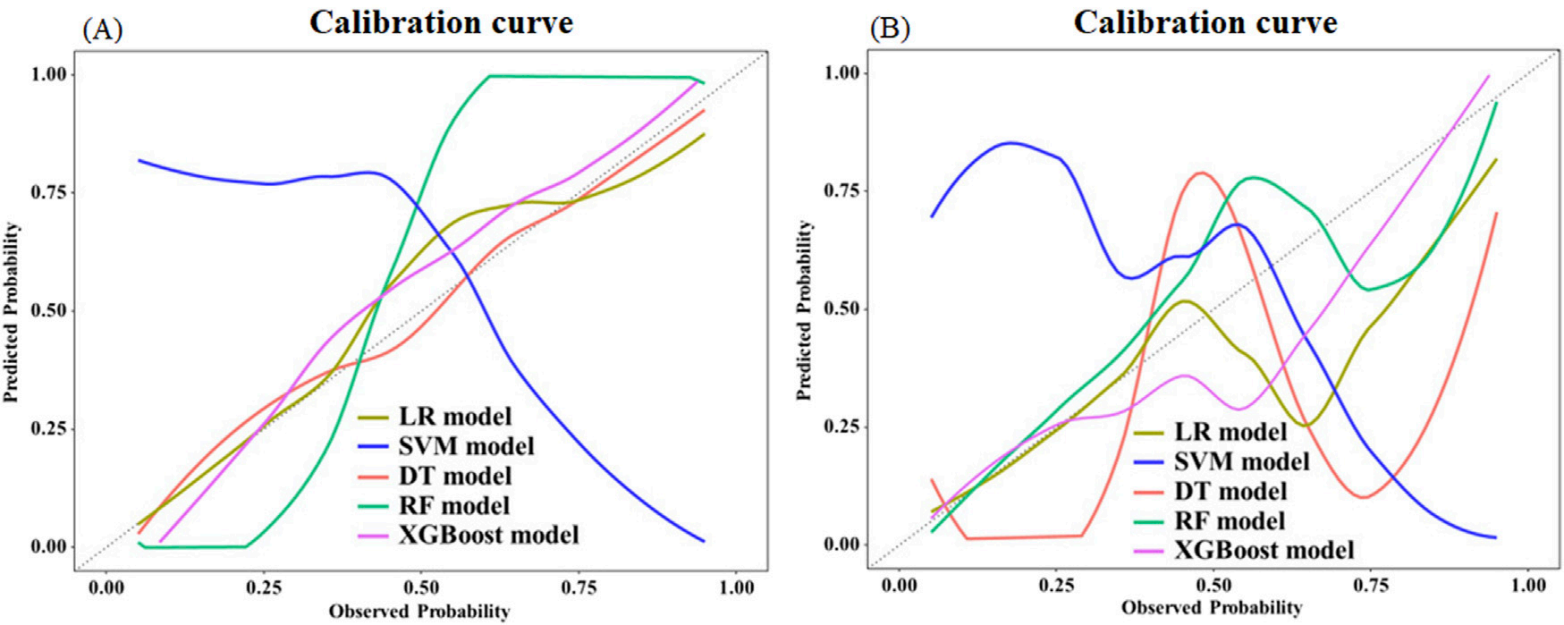
Comparison of calibration curves between different models in the training set **(A)** and test set **(B)**. The calibration curve plots the predicted probability of in-hospital mortality against the observed frequency. The dashed diagonal line represents a perfectly calibrated model. Closer proximity of a model’s curve to the diagonal indicates better calibration, meaning its predictions are more reliable and trustworthy.

In the training set, the DT model exhibited the most optimal calibration, succeeded by XGBoost, LR, RF, and SVM models. In the test set, the calibration performance ranked from highest to lowest as follows: XGBoost, RF, LR, DT, and SVM model (Figure 6).

#### 3.4.3 Clinical practicability

**FIGURE 7:**
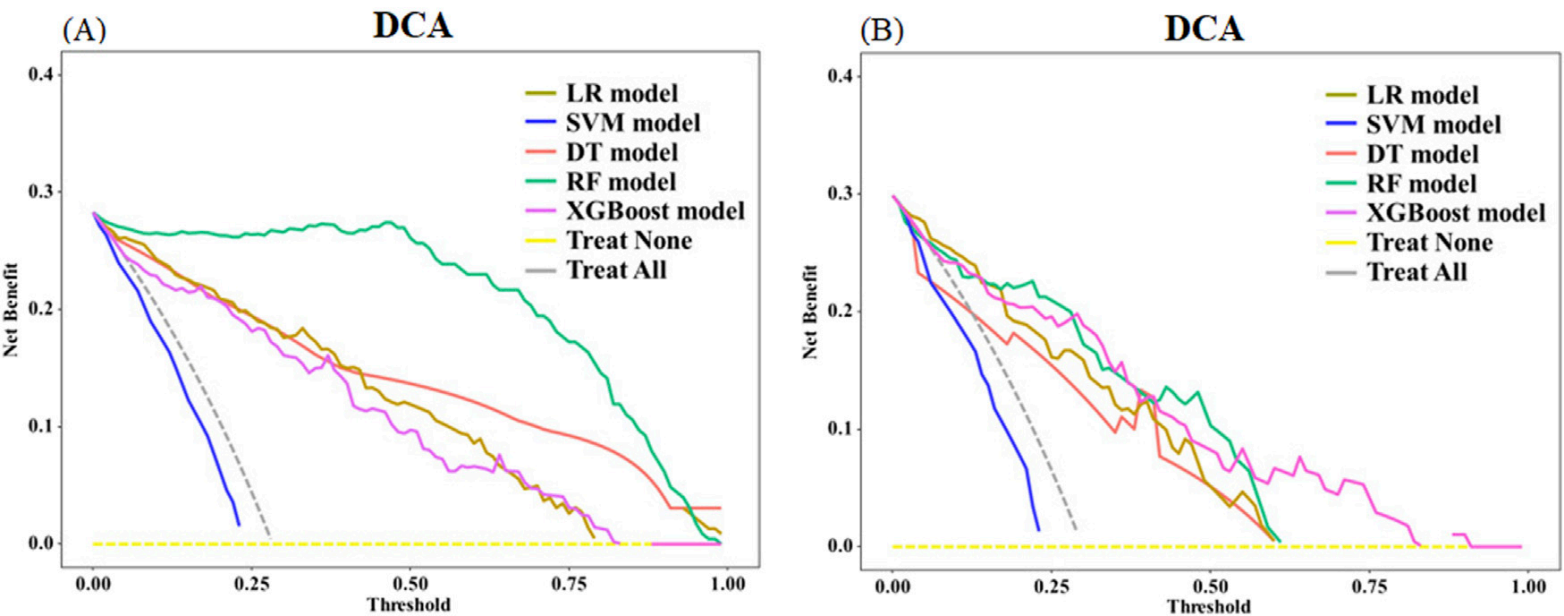
Comparison of DCA between different models in the training set **(A)** and test set **(B)**. X-axis indicates the threshold probability and Y-axis indicates the net benefit. The dashed gray line indicates that all severe pneumonia patients had hospital death, while the dashed yellow line indicates that no patient had hospital death.

In the training set, the net benefit of the RF model exceeded that of the DT, LR, XGBoost, and SVM models as indicated by the DCA. In the test set, the XGBoost model exhibited the highest net benefit, while the SVM model performed the poorest, indicating that the XGBoost model was the most optimal. Moreover, with the exception of the SVM model, DCA curves showed that the other four models demonstrate clinical value (Figure 7).

#### 3.4.4 The VIF analysis

The VIF values for seven predictors in our final LR model were all below 10 (analyzed using the car package, version 3.1-2, in R)), suggesting that multicollinearity was not a significant concern that would destabilize the model. The detailed results were shown in Table 4.

**TABLE 4:** The VIF values for seven predictors in our final LR model.

Predictor	VIF
Age	1.08
TCM syndrome (pathogenic qi falling into and prostration syndrome)	1.03
Complication of septic shock	1.06
BUN level	1.04
Tracheotomy application	1.06
Retention catheterization application	1.11
Oral Chinese herbal decoction	1.07

### 3.5 Optimal model analysis

The XGBoost model was selected as the optimal model based on its balanced and superior overall performance in discrimination (AUROC = 0.853), calibration, and clinical practicability (as evidenced by DCA) on the test set, compared to the other four models and traditional scoring systems.

**FIGURE 8:**
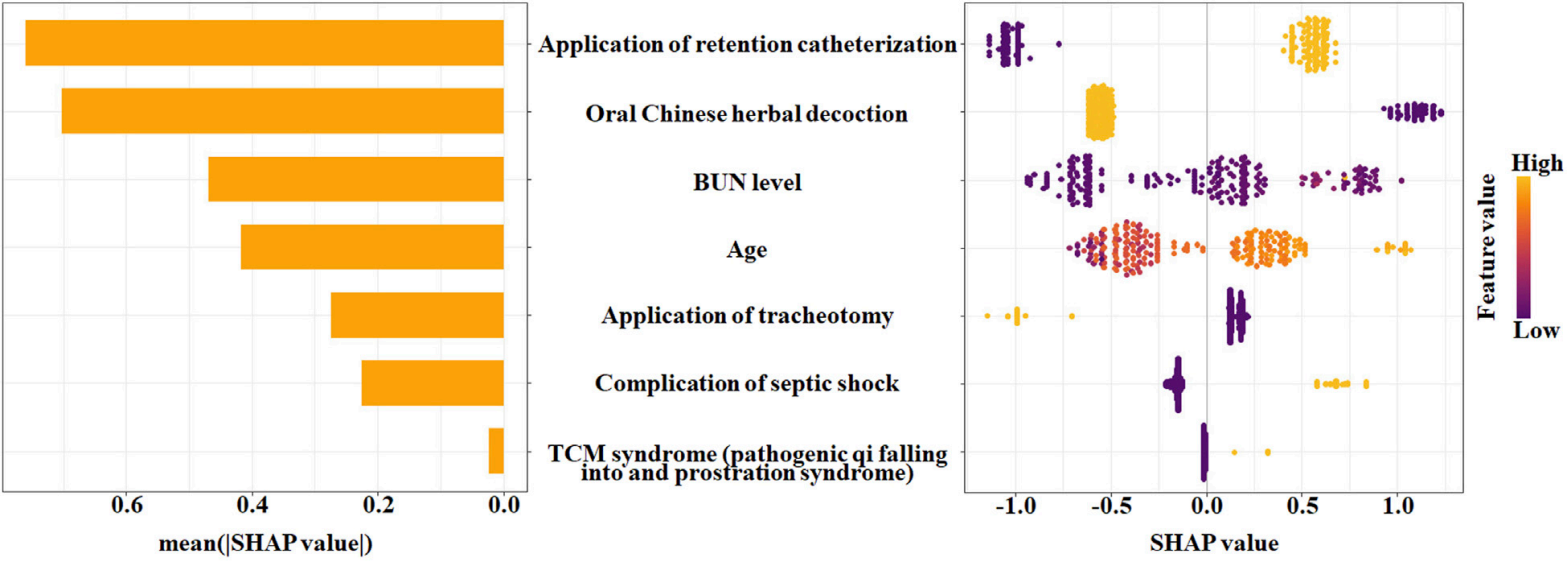
Interpretation of the optimal XGBoost model using SHAP values. SHAP values indicate the direction of the contribution of a single feature to the model output. Left: The bar plot shows the mean absolute SHAP value for each feature, representing its overall importance in the model. Right: The beeswarm plot shows the distribution of SHAP values for each feature for every patient in the test set. The x-axis indicates the SHAP value (impact on the model output), where a positive value increases the predicted risk of death. The color indicates the feature value for an individual patient (yellow: high value or presence of the factor; purple: low value or absence).

To interpret the XGBoost model and assess the contribution of each predictor, we calculated SHapley Additive exPlanations (SHAP) values on the test set. Figure 8 presents the summary plot of the SHAP values for the seven predictors selected by the LASSO regression. The mean absolute SHAP value (shown in the bar plot on the left) represents the average impact of each feature on the model output. The application of retention catheterization was identified as the most important influential predictor in the model, followed by oral Chinese herbal decoction, BUN level, age, application of tracheotomy, complication of septic shock, and TCM syndrome (pathogenic qi falling into and prostration syndrome). For each predictor, the scatter plot shows how its value affects the prediction: in the context of this model, higher values of BUN and age, as well as the presence of septic shock, retention catheterization, and the TCM syndrome (yellow dots), were generally associated with positive SHAP values, suggesting their role as risk factors in the decision process of model. Conversely, the application of tracheotomy and oral Chinese herbal decoction (yellow dots) were associated with negative SHAP values, indicating that these factors contributed to a lower predicted risk in the model’s output.

## 4 Discussion

### 4.1 Principal findings

Our study retrospectively gathered clinical data from 226 patients with severe pneumonia for the training set, of whom 64 died in-hospital, resulting in a mortality of 28.32%, consistent with findings from prior studies (Kassaw et al., 2023). The Lasso regression analysis was conducted to identify risk factors associated with severe pneumonia mortality in relation to Chinese and conventional medicine, including age, complication of septic shock, BUN level, TCM syndrome (pathogenic qi falling into and prostration syndrome), application of tracheotomy, retention catheterization, and oral Chinese herbal decoction. The implementation of tracheotomy and the administration of oral Chinese herbal decoction were strongly associated with improved mortality outcomes of patients with severe pneumonia. We constructed and validated models capable of predicting mortality in patients with severe pneumonia using routinely available clinical data, and compared five machine learning algorithms. The XGBoost model is superior to the overall performance of LR, SVM, DT, RF, as well as the scoring systems of PSI, SOFA, and APACHE II. The SHAP method explains the XGBoost model, so enhancing both model performance and clinical interpretability. This model may possess potential utility in personalized surveillance prognosis, facilitating improved therapy schedules and appropriate resource allocation for patients.

Machine learning is characterized by its applicability to various types of datasets, resulting in its widespread utilization. Nonetheless, various algorithms possess distinct benefits, and their capacity and efficacy in problem-solving mostly depend on the characteristics of data aspects and the performance of algorithms. Consequently, evaluating the efficacy of various machine learning algorithms on a particular dataset to identify the ideal model, together with employing feature importance analysis to enhance comprehension of presented features, is highly significant (Domaratzki and Kidane, 2022). The most significant predictive factor in the optimal XGBoost model is the application of retention catheterization, succeeded by oral Chinese herbal decoction, BUN level, age, tracheotomy application, complication of septic shock, and TCM syndrome (pathogenic qi falling into and prostration syndrome).

Patients with severe pneumonia frequently present with multiple underlying diseases, and those in critical condition often suffer from consciousness disorders, hindering their ability to urinate autonomously; thus, the application of retention catheterization is required. However, this study identified that indwelling catheters are an important risk factor for mortality due to severe pneumonia, corroborating findings from previous studies (Zhu et al., 2023).

Recent studies have demonstrated that TCM, when combined with conventional treatment, offers improvements in the management of severe pneumonia (Xie et al., 2023). Our research showed that a duration of over 5 days of oral Chinese herbal decoction might decrease the mortality risk of severe pneumonia, hence revealing the efficacy of TCM for treating severe pneumonia based on syndrome differentiation.

BUN is a primary end product of protein metabolism in the human body and serves as a crucial indication for assessing kidney function. The lung and kidney exhibit complex connections, both playing crucial organs in regulating acid-base and fluid balance (Hepokoski et al., 2018). In addition, any impairment to the kidneys can substantially impact the lungs by disturbing normal the pH and fluid distribution balance. Furthermore, the kidneys may promote the progression and regulate of pulmonary illnesses by the production or elimination of mediators. The interaction between the lungs and kidneys highlights their mutual dependence and impact on overall physiological function (Wang et al., 2021). A study indicated that patients with acute kidney injury and pneumonia exhibited a greater mortality compared to those with either condition alone (Chawla et al., 2017). Furthermore, a diagnostic criterion for severe pneumonia includes BUN levels, signifying a strong association between BUN and disease severity. Our study demonstrates that BUN is a significant risk factor for increased mortality in patients with severe pneumonia, potentially attributable to the relationship between the lung microbiome in these patients and kidney damage (Du et al., 2022).

With advancing age, the human immune system experiences various alterations, resulting in diminished capacity to efficiently trigger cellular responses against pathogens. The chemotactic capacity of polymorphonuclear leukocytes in the elderly is weakened, and the microbial uptake and antigen processing capabilities of macrophages are correspondingly reduced (Mahendra et al., 2018). Moreover, age-related factors such as chronic comorbidities, alterations in immunological physiology, and malnutrition substantially increase the risk of infection in the elderly (Arvaniti et al., 2022). The results of this study indicate that the risk of mortality from severe pneumonia increases with age, which is consistent with previous research findings (Li Y. et al., 2021) and potentially linked to age-associated chronic disorders and/or diminished immune function (Wang et al., 2020).

Individuals with severe pneumonia display a substantial elevation in airway secretions. When accompanied with consciousness problems, severe cerebral infarction, traumatic brain injury, or additional problems, respiratory function becomes impaired, requiring ventilator support. Elderly patients, due to their numerous medical conditions, are susceptible to difficulties such as the accumulation of airway secretions, respiratory obstruction, and throat injury during prolonged laryngotracheal intubation, potentially resulting in complications such ventilator-associated pneumonia (Kaese et al., 2016). Therefore, for severe pneumonia patients on prolonged ventilator support with stable conditions, tracheotomy may be considered if extended ventilatory assistance is essential. Our study found that tracheotomy serves as a preventive factor against mortality associated with severe pneumonia, significantly reducing the risk of death. Nonetheless, owing to limitations in clinical data collection, the precise best present moment for incision requires additional investigation.

Pneumonia is the major cause of septic shock, responsible for 50% of cases (Guzzardella et al., 2023; Spencer et al., 2022; Güell et al., 2019). A retrospective clinical survey of 710 patients indicated that the mortality for individuals with severe pneumonia complicated with septic shock was greater than for those without septic shock (Güell et al., 2019). Our study identified concurrent septic shock as a significant risk factor for increased mortality in patients with severe pneumonia, corroborating findings from prior research (Espinoza et al., 2019) and mutually confirming that septic shock is one of the two primary diagnostic criteria for severe pneumonia (Ferrer et al., 2018).

TCM syndromes serve as significant indicators for disease progression, aiding in the prognostic assessment of patients according to their syndrome classifications or developments (Lu and Deng, 2017). Previous studies have shown that common symptoms of severe pneumonia include phlegm-heat obstructing lung syndrome, deficiency of both qi and yin syndrome, pathogenic qi falling into and prostration syndrome, and phlegm turbidity obstructing lung syndrome (Zhang et al., 2022). Our study identified that the pathogenic qi falling into and prostration syndrome were risk factors for mortality in severe pneumonia, with the presence of this syndrome frequently indicating a fatal outcome.

Furthermore, the distribution of cases across different solar terms showed a significant difference between the training and test sets. While the clinical relevance of this TCM-based temporal variable requires further investigation, it highlights the potential influence of seasonal climatic factors, as conceptualized in TCM, on the presentation or course of severe pneumonia.

### 4.2 Strengths compared to previous constructed models

Currently, multiple predictive models exist concerning the mortality risk associated with severe pneumonia. We did a thorough search and systematic comparison, revealing that the model we developed possesses particular characteristics and advantages. A recent study developed an ensemble machine learning model for in-hospital mortality in severe pneumonia, reporting a competitive AUC of 0.878 in their internal validation set (Zhao et al., 2025), which is comparable to our finding (AUC = 0.853). We note that their model, while performing excellently, was derived from a single-center retrospective cohort. Our study complements this by incorporating TCM characteristics and employing prospective validation for our test set, enhancing the clinical applicability and uniqueness of our model. A study established the LR, gradient-boosted decision tree (LightGBM), and multilayer perceptron (MLP) models to forecast ICU mortality in patients with severe pneumonia (Pan et al., 2023). The best MLP model achieved an AUC of 0.838, which was inferior than the AUC of 0.853 obtained by our XGBoost model. Three studies constructed multivariable LR models with an AUC of 0.836 (Shang et al., 2023), 0.728 (Zhu et al., 2021) and 0.76 (Niu et al., 2025) for predicting in-hospital mortality in elderly patients with severe community-acquired pneumonia (SCAP). Additionally, another LR model, which lacked validation, reported an AUC as high as 0.915 in the training set (Zhu et al., 2021). A study (Pan et al., 2023) exclusively employed the LR method rather than machine learning algorithms to develop an in-hospital mortality risk prediction model for patients with SCAP. An alternative LR model predicting 30-day mortality in ICU patients with SCAP exhibited a lower AUC of 0.756 (Zhang Y. et al., 2023). Nevertheless, our research additionally produced four models: SVM, DT, RF, and XGBoost, with the performance of our best model superior than that of models developed in prior studies.

In summary, the model we developed possesses the following advantages: First of all, we employed multiple algorithms for machine learning, including SVM, DT, RF, and XGBoost, rather than solely relying on LR, and identified the optimal XGBoost model. Secondly, the optimal model we have developed exhibits markedly superior discrimination compared to previously published models, with an AUC of 0.853. Thirdly, and most importantly, prior models failed to incorporate TCM features, whereas our study first gathered 115 clinical features. Among the seven risk factors linked to in-hospital mortality in severe pneumonia identified by LASSO regression, two were TCM factors: TCM syndrome (pathogenic qi dropping into and prostration syndrome) and oral Chinese herbal decoction. Consequently, our model could provide a more comprehensive review of the severe pneumonia patients state and yield reliable predictions.

### 4.3 Limitations

Our study has several limitations that should be acknowledged. Firstly, the retrospective design and relatively limited sample size may introduce risks of unmeasured confounding and missing data bias, despite comprehensive quality control measures. Furthermore, the observed overfitting of the RF model underscores the challenges of model complexity in the context of limited sample size and a substantial number of features. Secondly, our observational design is susceptible to indication bias and unmeasured confounding. Specifically, the variable “oral Chinese herbal decoction” was defined as a binary indicator of whether a patient received any professionally prescribed and hospital-dispensed decoction for a minimum duration during hospitalization. Consistent with standard TCM practice, these decoctions were not standardized but were individualized based on pattern differentiation by certified practitioners. Consequently, the specific herbal composition, dosage, and treatment duration varied between patients. While this reflects real-world clinical application, it means the variable captures a complex intervention, and its observed association with improved survival may reflect its preferential administration to less severely ill patients capable of oral intake, rather than a direct causal effect. Although we adjusted for multiple measures of disease severity, residual confounding cannot be fully excluded. Thirdly, the generalizability of the model may be constrained by its development within a TCM-oriented healthcare context. All data originated from two TCM-affiliated hospitals in China. The distribution of patients between these two centers was uneven in both the training and test sets, with one center (First Affiliated Hospital of Henan University of Chinese Medicine) contributing a larger share of patients. Although our model performed well on the test set which contained a higher proportion of patients from the smaller center (Henan Provincial Hospital of Chinese Medicine), suggesting some robustness to inter-hospital variation, the potential for center-specific bias cannot be entirely ruled out. Therefore, the performance of our model in other geographic regions, or more balanced multi-center settings remains to be determined. External validation in broader, more diverse populations is strongly recommended. Fourthly, our machine learning models were compared using their default hyperparameters. While this approach enhances clinical practicality and reduces the risk of overfitting on our dataset, it may not represent the fully optimized potential of each algorithm. Future studies with larger cohorts could incorporate advanced hyperparameter tuning techniques to further maximize predictive performance. Lastly, although we compared five commonly used machine learning algorithms, other methods were not included. Further investigations could explore a wider range of modeling strategies.

## 5 Conclusion

Older age, increased BUN level, complication of septic shock, tracheotomy application, retention catheterization application, oral Chinese herbal decoction, and TCM syndrome (pathogenic qi falling into and prostration syndrome) may be potential risk factors that affect mortality in severe pneumonia, while application of tracheotomy and oral Chinese herbal decoction were associated with reduced mortality. The XGBoost model exhibits superior overall performance in predicting hospital mortality risk for severe pneumonia, greater than traditional scoring systems such as PSI, SOFA, and APACHE II, which assists clinicians in prognostic assessment, resulting in improved therapeutic strategies and optimal resource allocation for patients.

## Data Availability

The original contributions presented in the study are included in the article/Supplementary Material, further inquiries can be directed to the corresponding author.

## Glossary

ICU: intensive care unit
CRP: C-reactive protein
IL: interleukin
mNUTRIC: Modified Nutrition Risk in Critically ill
TCM: traditional Chinese medicine
AI: artificial intelligence
SOFA: Sequential Organ Failure Assessment
APACHE II: Acute Physiology and Chronic Health Evaluation II
LR: Logistic Regression
SVM: Support Vector Machine
DT: Decision Tree
RF: Random Forest
XGBoost: Extreme Gradient Boosting
H1N1: Influenza A
SARS: severe acute respiratory syndrome
COVID-19: coronavirus disease 2019
COPD: chronic obstructive pulmonary disease
WBC: white blood cell
RBC: red blood cell
NEUT%: neutrophilic granulocyte percentage
LY%: lymphocyte percentage
PCT: procalcitonin
AST: aminotransferase
ALT: alanine aminotransferase
BUN: blood urea nitrogen
Scr: serum creatinine
PT: prothrombin time
BNP: brain natriuretic peptide
PaO2: arterial partial pressure of oxygen
PaCO2: arterial partial pressure of carbon dioxide
PaO2/FiO2: arterial oxygenation index
SD: standard deviation
IQR: interquartile range
LASSO: Least Absolute Shrinkage and Selection Operator
AUROC: area under the receiver operating characteristic
MCC: matthew correlation coefficient
ROC: receiver operation characteristic
AUC: area under the curve
DCA: decision curve analysis
PSI: Pneumonia Severity Index
SHAP: SHapley Additive exPlanations
LightGBM: gradient-boosted decision tree
MLP: multilayer perceptron
SCAP: severe community acquired pneumonia.
