# Supplementary material for: Development and validation of a clinical prediction model for in-hospital mortality of severe pneumonia based on machine learning
Source: Front Pharmacol. 2025 Nov 26;16:1660893. doi: 10.3389/fphar.2025.1660893 (PMC12689516; doi:10.3389/fphar.2025.1660893)
Supplement: Supplementary file 1 [file Table1.docx]

**Supplements**

**Supplementary Material 1** **Detailed parameter specifications for machine learning models**

Table S1 Detailed parameter specifications for machine learning models

| Algorithm | R Package (Version) | Core parameters and values |
| --- | --- | --- |
| LR | stats (base R, v4.3.2) | family = binomial (logit) |
| SVM | e1071 (1.7-13) | kernel = "linear", probability = TRUE |
| DT | rpart (4.1.19) | method = "class", parms = list (split="information") |
| RF | randomForest (4.7-1.1) | ntree = 500, mtry = sqrt (p), nodesize = 1, maxdepth = unlimited |
| XGBoost | xgboost (1.7.5) | nrounds = 1000, max_depth = 2, eta = 0.3, subsample = 0.7, colsample_bytree = 0.4, early_stopping_rounds = 200 |

Notes: All analyses were conducted using R version 4.3.2. For RF, p represents the number of predictors in the dataset.

**Supplementary Material 2 Complete dataset of all features compared between non-survivors and survivors in the training set.**

In the training set, there were significant statistical differences between the non-survivors and survivors in a total of 38 factors including age, TCM syndrome, risk factors before admission of fracture history, long-term bed rest history, and intravenous antibiotics within 30 days, comorbidities for cerebral infarction, cardiac insufficiency, gastrointestinal bleeding, and cancer, complications for electrolyte imbalance, anemia, hypoproteinemia, pleural effusion, and septic shock, laboratory results for hematokrit, NEUT%, PCT, total protein, albumin, BUN, Scr, troponin, myohemoglobin, fibrinogen, D-dimer, and arterial blood PH, as well as antifungal drug, transfusion, tracheotomy, retention catheterization, gastric intubation, deep vein catheterization, days of nasal tube oxygen, days of invasive mechanical ventilation, days of mechanical ventilation, oral Chinese herbal decoctions, TCM syndrome, total hospitalization days, days of ICU stay (*P* < 0.05), which might be the potential risk factors for death in patients with severe pneumonia. The detailed was presented in Table S2.

Table S2 Features comparison between the non-survivors and the survivors for severe pneumonia patients in the training set

| Feature | Non-survivors  (*n*=64) | Survivors  (*n*=162) | *P value* |
| --- | --- | --- | --- |
| **Age** | 78.5 (69.25-85.75) | 70.5 (58.75-79.25) | <0.001* |
| **Male** | 44 (68.75%) | 114 (70.37%) | 0.938 |
| **Nationalitiy** |  |  | 1.000 |
| Han | 64 (100.00%) | 160 (98.77%) |  |
| others | 0 (0.00%) | 2 (1.23%) |  |
| **Solar term** |  |  | 0.319 |
| Lesser Cold | 2 (3.13%) | 14 (8.64%) |  |
| Greater Cold | 2 (3.13%) | 14 (8.64%) |  |
| the Beginning of Spring | 2 (3.13%) | 7 (4.32%) |  |
| Rain Water | 2 (3.13%) | 8 (4.94%) |  |
| Insects awaken | 3 (4.69%) | 6 (3.70%) |  |
| the Spring Equinox | 2 (3.13%) | 4 (2.47%) |  |
| Pure Brightness | 1 (1.56%) | 6 (3.70%) |  |
| Grain Rain | 3 (4.69%) | 7 (4.32%) |  |
| the Beginning of Summer | 1 (1.56%) | 6 (3.70%) |  |
| Lesser Fullness of Grain | 1 (1.56%) | 4 (2.47%) |  |
| Grain in Beard | 6 (9.38%) | 10 (6.17%) |  |
| the Summer Solstice | 3 (4.69%) | 3 (1.85%) |  |
| Lesser Heat | 2 (3.13%) | 9 (5.56%) |  |
| Greater Heat | 3 (4.69%) | 8 (4.94%) |  |
| the Beginning of Autumn | 3 (4.69%) | 3 (1.85%) |  |
| the End of Heat | 2 (3.13%) | 5 (3.09%) |  |
| White Dew | 0 (0.00%) | 5 (3.09%) |  |
| the Autumn Equinox | 7 (10.94%) | 4 (2.47%) |  |
| Cold Dew | 2 (3.13%) | 6 (3.70%) |  |
| Frost’s Descent | 2 (3.13%) | 6 (3.70%) |  |
| the Beginning of Winter | 4 (6.25%) | 7 (4.32%) |  |
| Lesser Snow | 0 (0.00%) | 7 (4.32%) |  |
| Greater Snow | 4 (6.25%) | 6 (3.70%) |  |
| the Winter Solstice | 7 (10.94%) | 7 (4.32%) |  |
| **Vital signs** |  |  |  |
| Body temperature (℃) | 37.05 (36.5-38) | 36.9 (36.5-38.3) | 0.903 |
| Respiratory rate (breaths/min) | 22.5 (20-30) | 21 (20-25) | 0.301 |
| Heart rate (beats/min) | 100.5 (84.25-116.75) | 95.5 (80-112) | 0.385 |
| Systolic pressure (mmHg) | 126 (108.5-145.75) | 126 (116-140) | 0.779 |
| Diastolic pressure (mmHg) | 76 (65-84) | 77 (70-84) | 0.363 |
| **Risk factors before admission** |  |  |  |
| Allergic history | 8 (12.50%) | 19 (11.73%) | 1.000 |
| Smoking history | 10 (15.63%) | 36 (22.22%) | 0.281 |
| Alcohol consumption history | 7 (10.94%) | 28 (17.28%) | 0.308 |
| Fracture history | 12 (18.75%) | 13 (8.02%) | 0.032* |
| Surgery history | 28 (43.75%) | 55 (33.95%) | 0.220 |
| Long-term bed rest history | 35 (54.69%) | 62 (38.27%) | 0.026* |
| Hospitalization within 90 days | 38 (59.38%) | 99 (61.11%) | 0.880 |
| ICU admission within 90 days | 7 (10.94%) | 34 (20.99%) | 0.087 |
| Intravenous antibiotics within 30 days | 23 (35.94%) | 89 (54.94%) | 0.010* |
| Dialysis within 30 days | 0 (0.00%) | 7 (4.32%) | 0.207 |
| **Comorbidities** |  |  |  |
| Hypertension | 36 (56.25%) | 81 (50.00%) | 0.461 |
| Diabetes | 20 (31.25%) | 48 (29.63%) | 0.872 |
| Chronic bronchitis | 10 (15.63%) | 18 (11.11%) | 0.374 |
| COPD | 12 (18.75%) | 21 (12.96%) | 0.298 |
| Pulmonary fibrosis | 8 (12.50%) | 19 (11.73%) | 1.000 |
| Bronchiectasis | 1 (1.56%) | 6 (3.70%) | 0.681 |
| Asthma | 1 (1.56%) | 9 (5.56%) | 0.339 |
| Old pulmonary tuberculosis | 1 (1.56%) | 8 (4.94%) | 0.428 |
| Pulmonary abscess | 0 (0.00%) | 2 (1.23%) | 1.000 |
| Pulmonary heart disease | 2 (3.12%) | 8 (4.94%) | 0.812 |
| Arrhythmia | 21 (32.81%) | 33 (20.37%) | 0.057 |
| Cardiac insufficiency | 12 (18.75%) | 10 (6.17%) | 0.006* |
| Chronic heart failure | 10 (15.63%) | 13 (8.02%) | 0.140 |
| Parkinson’s disease | 3 (4.69%) | 7 (4.32%) | 1.000 |
| Cerebral infarction | 35 (54.69%) | 52 (32.10%) | 0.002* |
| Hematencephalon | 8 (12.50%) | 15 (9.26%) | 0.626 |
| Chronic gastritis | 5 (7.81%) | 7 (4.32%) | 0.468 |
| Gastrointestinal bleeding | 6 (9.38%) | 2 (1.23%) | 0.010* |
| Chronic viral hepatitis | 2 (3.12%) | 10 (6.17%) | 0.554 |
| Liver cirrhosis | 1 (1.56%) | 6 (3.70%) | 0.681 |
| Chronic renal insufficiency | 4 (6.25%) | 5 (3.09%) | 0.473 |
| Chronic renal failure | 2 (3.12%) | 6 (3.70%) | 1.000 |
| Cancer | 9 (14.06%) | 5 (3.09%) | 0.005* |
| Lumbar disease | 6 (9.38%) | 9 (5.56%) | 0.458 |
| Neck disease | 2 (3.12%) | 4 (2.47%) | 1.000 |
| **Complications** |  |  |  |
| Acid base disturbance | 36 (56.25%) | 88 (54.32%) | 0.882 |
| Electrolyte imbalance | 48 (75.00%) | 97 (59.88%) | 0.045* |
| Anemia | 46 (71.88%) | 89 (54.94%) | 0.024* |
| Hypoproteinemia | 59 (92.19%) | 130 (80.25%) | 0.029* |
| Pleural effusion | 62 (96.88%) | 134 (82.72%) | 0.005* |
| Acute myocardial infarction | 3 (4.69%) | 4 (2.47%) | 0.659 |
| Acute heart failure | 9 (14.06%) | 10 (6.17%) | 0.065 |
| Acute kidney injury | 12 (18.75%) | 7 (4.32%) | 0.389 |
| Acute liver injury | 7 (10.94%) | 13 (8.02%) | 0.603 |
| Hypovolemic shock | 2 (3.12%) | 2 (1.23%) | 0.681 |
| Septic shock | 18 (28.13%) | 11 (6.79%) | <0.001* |
| Cardiac shock | 2 (3.12%) | 4 (2.47%) | 1.000 |
| **Laboratory results** |  |  |  |
| WBC (×10^9^/L) | 9.85 (6.66-15.71) | 8.6 (6.68-12.03) | 0.187 |
| RBC (×10¹²/L) | 3.68 (3.1-4.32) | 3.91 (3.41-4.43) | 0.087 |
| Hemoglobin (g/L) | 111 (94.25-129.5) | 119 (101.75-134.25) | 0.061 |
| Hematokrit (%) | 33.9 (28.75-38.38) | 36.6 (31.35-40.73) | 0.021* |
| Platelet count (×10^9^/L) | 184.5 (112-236) | 191 (134.75-249.5) | 0.214 |
| NEUT% | 88.7 (82.95-93.2) | 84.2 (76.28-89.83) | 0.004* |
| LY% | 7.55 (3.87-12.38) | 9.85 (5.93-15.1) | 0.076 |
| CRP (mg/L) | 84.11 (35.5-161.91) | 78.79 (30.92-161) | 0.449 |
| PCT (μg/L) | 0.62 (0.36-2.95) | 0.35 (0.1-0.77) | <0.001* |
| Total bilirubin (μmol/L) | 15.15 (9.93-24.43) | 12.85 (9.3-18.9) | 0.100 |
| Total protein (g/L) | 56.71±11.19 | 60.44±8.88 | 0.019* |
| Albumin (g/L) | 29.75±6.3 | 32.19±4.93 | 0.007* |
| ALT (U/L) | 22.05 (13.25-35.68) | 22.1 (13.45-39.53) | 0.920 |
| AST (U/L) | 30.25 (19.28-58.25) | 26.25 (16.98-44.1) | 0.281 |
| BUN (mmol/L) | 11.85 (7.4-17.52) | 6.48 (4.63-10.38) | <0.001* |
| Scr (μmol/L) | 81.75 (56-134.58) | 65.5 (50.15-94.7) | 0.021* |
| Potassium (mmol/L) | 4.2±0.76 | 4.15±0.75 | 0.654 |
| Sodium (mmol/L) | 137.2 (132.1-141.78) | 137.6 (134.88-141) | 0.619 |
| Troponin (ng/ml) | 0.05 (0.05-0.19) | 0.05 (0.01-0.07) | <0.001* |
| Myohemoglobin (ng/ml) | 40.74 (40.74-78) | 40.74 (21.1-69.83) | 0.022* |
| PT (s) | 13.75 (12.2-15.58) | 12.95 (11.7-14.53) | 0.051 |
| APTT (s) | 34.55 (29.3-40.25) | 33.05 (28.48-39.28) | 0.253 |
| Fibrinogen (g/L) | 4.62 (3.03-5.8) | 5.37 (4.1-6.56) | 0.013* |
| D-dimer (μg/ml) | 2.93 (1.79-5.06) | 2.11 (1.02-3.79) | 0.007* |
| BNP (pg/ml) | 190 (80.16-578.63) | 245 (83.96-881.5) | 0.392 |
| Arterial blood PH | 7.43 (7.36-7.46) | 7.44 (7.42-7.48) | 0.008* |
| PaO_2_ (mmHg) | 61.4 (56-83.8) | 61.4 (54-70.08) | 0.174 |
| PaCO_2_ (mmHg) | 32.95 (25.38-37.95) | 32.95 (29.95-40.08) | 0.196 |
| PaO_2_/FiO_2_ | 229.5 (171.75-261.75) | 229.5 (193.75-276) | 0.101 |
| **Application of conventional medicine** |  |  |  |
| Glucocorticoids | 36 (56.25%) | 91 (56.17%) | 1.000 |
| Number of antibiotics ≥3 | 54 (84.38%) | 117 (72.22%) | 0.055 |
| Beta-lactam antibiotics | 64 (100.00%) | 158 (97.53%) | 0.479 |
| Quinolone antibiotics | 41 (64.06%) | 122 (75.31%) | 0.101 |
| Aminoglycoside antibiotics | 9 (14.06%) | 17 (10.49%) | 0.490 |
| Macrolide antibiotics | 12 (18.75%) | 25 (15.43%) | 0.553 |
| Tetracycline antibiotics | 16 (25.00%) | 24 (14.81%) | 0.083 |
| Sulfonamide antibiotics | 0 (0.00%) | 1 (0.62%) | 1.000 |
| Antifungal drug | 27 (42.19%) | 45 (27.78%) | 0.041* |
| Immunosuppressant | 4 (6.25%) | 5 (3.09%) | 0.473 |
| **Conventional operation** |  |  |  |
| Fiber bronchoscope | 31 (48.44%) | 76 (46.91%) | 0.883 |
| Transfusion | 21 (32.81%) | 31 (19.14%) | 0.035* |
| Hemodialysis | 4 (6.25%) | 8 (4.94%) | 0.947 |
| ECMO | 1 (1.56%) | 1 (0.62%) | 0.487 |
| Tracheotomy | 3 (4.69%) | 30 (18.52%) | 0.008* |
| Retention catheterization | 56 (87.50%) | 73 (45.06%) | <0.001* |
| Gastric intubation | 45 (70.31%) | 78 (48.15%) | 0.003* |
| Deep vein catheterization | 39 (60.94%) | 75 (46.30%) | 0.047* |
| Days of nasal tube oxygen | 0 (0-8.5) | 4 (0-14) | 0.019* |
| Days of mask oxygen days | 0 (0-1) | 0 (0-0) | 0.095 |
| Days of non-invasive mechanical ventilation | 0 (0-3) | 0 (0-2) | 0.098 |
| Days of invasive mechanical ventilation | 1.5 (0-8) | 0 (0-4) | <0.001* |
| Days of mechanical ventilation | 6.5 (1-11) | 0 (0-11) | 0.005* |
| **TCM or TCM appropriate technology** |  |  |  |
| Oral Chinese herbal decoction | 27 (42.19%) | 139 (85.80%) | <0.001* |
| Chinese patent medicine injection | 55 (85.94%) | 126 (77.78%) | 0.198 |
| TCM appropriate technology | 54 (84.38%) | 136 (83.95%) | 1.000 |
| **TCM syndrome** |  |  | <0.001* |
| Phlegm-heat obstructing lung syndrome | 13 (20.31%) | 65 (40.12%) |  |
| Phlegm turbidity obstructing lung syndrome | 17 (26.56%) | 26 (16.05%) |  |
| Deficiency of both qi and yin syndrome | 9 (14.06%) | 14 (8.64%) |  |
| Lung-spleen qi deficiency syndrome | 6 (9.38%) | 10 (6.17%) |  |
| Lung-spleen qi deficiency combined with phlegm turbidity obstructing lung syndrome | 2 (3.13%) | 11 (6.79%) |  |
| Phlegm turbidity obstructing lung combined with stagnation of blood syndrome | 2 (3.13%) | 7 (4.32%) |  |
| Deficiency of both qi and yin combined with phlegm turbidity obstructing lung syndrome | 1 (1.56%) | 7 (4.32%) |  |
| Pathogenic qi falling into and prostration syndrome | 6 (9.38%) | 1 (0.62%) |  |
| Invasion of pericardium by heat syndrome | 0 (0.00%) | 6 (3.70%) |  |
| Phlegm-heat obstructing lung combined with stagnation of blood syndrome | 1 (1.56%) | 5 (3.09%) |  |
| Deficiency of both qi and yin combined with phlegm-heat obstructing lung syndrome | 2 (3.13%) | 4 (2.47%) |  |
| Lung-spleen qi deficiency combined with phlegm-heat obstructing lung syndrome | 1 (1.56%) | 4 (2.47%) |  |
| Lung-spleen qi deficiency combined with stagnation of blood syndrome | 2 (3.13%) | 2 (1.23%) |  |
| Stagnation of blood syndrome | 1 (1.56%) | 0 (0.00%) |  |
| Deficiency of both qi and yin combined with stagnation of blood syndrome | 1 (1.56%) | 0 (0.00%) |  |
| **Others** |  |  |  |
| Multi-drug resistant bacterial infection | 20 (31.25%) | 49 (30.25%) | 1.000 |
| Total hospitalization days | 12 (8.25-23) | 17 (13-27) | 0.001* |
| Days of ICU stay | 4 (0-10.5) | 0 (0-4.25) | <0.001* |
